# Analysis of immunoinfiltration and EndoMT based on TGF-β signaling pathway-related genes in acute myocardial infarction

**DOI:** 10.1038/s41598-024-55613-5

**Published:** 2024-03-02

**Authors:** Jun Shen, Junqing Liang, Manzeremu Rejiepu, Zhiqin Ma, Jixian Zhao, Jia Li, Ling Zhang, Ping Yuan, Jianing Wang, Baopeng Tang

**Affiliations:** 1 Cardiac Pacing and Electrophysiology Department, The First Affiliated Hospital of Xinjiang Medical University, Urumqi, China; 2 Department of Cardiology, Renmin Hospital, Hubei University of Medicine, Shiyan, China

**Keywords:** Computational biology and bioinformatics, Cardiology

## Abstract

Acute myocardial infarction (AMI), a critical manifestation of coronary heart disease, presents a complex and not entirely understood etiology. This study investigates the potential role of immune infiltration and endothelial-mesenchymal transition (EndoMT) in AMI pathogenesis. We conducted an analysis of the GSE24519 and MSigDB datasets to identify differentially expressed genes associated with the TGF-β signaling pathway (DE-TSRGs) and carried out a functional enrichment analysis. Additionally, we evaluated immune infiltration in AMI and its possible link to myocardial fibrosis. Key genes were identified using machine learning and LASSO logistic regression. The expression of MEOX1 in the ventricular muscles and endothelial cells of Sprague–Dawley rats was assessed through RT-qPCR, immunohistochemical and immunofluorescence assays, and the effect of MEOX1 overexpression on EndoMT was investigated. Our study identified five DE-TSRGs, among which MEOX1, SMURF1, and SPTBN1 exhibited the most significant associations with AMI. Notably, we detected substantial immune infiltration in AMI specimens, with a marked increase in neutrophils and macrophages. MEOX1 demonstrated consistent expression patterns in rat ventricular muscle tissue and endothelial cells, and its overexpression induced EndoMT. Our findings suggest that the TGF-β signaling pathway may contribute to AMI progression by activating the immune response. MEOX1, linked to the TGF-β signaling pathway, appears to facilitate myocardial fibrosis via EndoMT following AMI. These novel insights into the mechanisms of AMI pathogenesis could offer promising therapeutic targets for intervention.

## Introduction

Acute myocardial infarction (AMI) is characterized by myocardial necrosis triggered by acute ischemia, leading to severe cardiac complications such as arrhythmias, heart failure, and sudden cardiac death^1,2^. AMI instigates a cascade of alterations in the myocardium, encompassing cardiomyocyte death, cardiac fibrosis, hypertrophy, and chamber dilation^3,4^. Cardiac fibrosis, a prevalent characteristic of failing hearts, is distinguished by myofibroblast differentiation and excessive accumulation of extracellular matrix (ECM) proteins, which can culminate in heart failure and increased mortality^5,6^. Elucidating the complex pathways involved in fibrotic signaling is crucial for the development of targeted therapies to mitigate cardiac fibrosis.

The transforming growth factor β (TGF-β) signaling pathway plays a pivotal role in the pathogenesis of cardiac fibrosis^7–9^.Although its critical function in regulating the phenotypes of fibroblasts and cardiomyocytes is well recognized, the intricacies of the TGF-β signaling cascade are not fully elucidated. The modulation of this pathway predominantly transpires through the mislocalization of TGF-β cytokines, receptors, and transcriptional factors, influenced by membrane and intracellular trafficking pathways^10–12^. MEOX1, a transcription factor, has been identified as being expressed in activated fibroblasts and plays a pivotal role in TGF-β-induced fibroblast activation^13,14^. Furthermore, the upregulation of MEOX1 following transverse aortic constriction (TAC) has been shown to accelerate myocardial hypertrophic decompensation via Gata4^15^. These observations implicate MEOX1 in the development of myocardial fibrosis during chronic heart failure^16^. However, a comprehensive understanding of MEOX1's expression and biological function in the context of acute myocardial infarction is lacking, particularly concerning its role in immune infiltration and endothelial-mesenchymal transition following myocardial infarction.

In this study, we aim to elucidate the molecular mechanisms of MEOX1 in the context of AMI by integrating data from the Gene Expression Omnibus (GEO) dataset with in vitro experimental findings. We procured a publicly available microarray dataset (GSE24519) pertinent to AMI and conducted a bioinformatics analysis to identify differentially expressed genes (DEGs). Among these, we pinpointed 55 genes associated with the TGF-β signaling pathway (TSRGs) and determined the intersecting genes between the DEGs and TSRGs. Through bioinformatics enrichment, we identified the key pathways and proteins associated with these overlapping genes. We also investigated the association between the expression of overlapping genes and immune cell infiltration, identified potential diagnostic biomarkers, and investigated the role of MEOX1 in endothelial-mesenchymal transition (EndoMT) during AMI. Our study provides valuable insights into the dysregulated expression of TGF-β signaling pathway-related genes and the potential mechanisms of immune infiltration in the pathogenesis of AMI, thereby suggesting novel targets for therapeutic intervention.

## Materials and methods

### Dataset acquisition and preprocessing

The microarray dataset GSE24519 was acquired from the GEO database (https://www.ncbi.nlm.nih.gov/geo/). GSE GSE24519, which consists of 34 cases of AMI and 4 control cases, was obtained from the GPL2895 sequencing platform. Principal Component Analysis (PCA) clustering was employed to calculate and generate overlapping or predefined clusters. Heat maps and volcano plots of DEGs were generated using the "heatmap" and "ggplot2" packages. A total of 55 genes related to the TGF-β signaling pathway were obtained from the MSigDB (HALLMARK_TGF_BETA_SIGNALING)^17^ and PubMed^13^.

### Analysis of differentially expressed TGF-β signaling pathway-related genes (DE-TSRGs)

The microarray series matrix files were imported into R for differential expression analysis, comparing normal and AMI samples using the limma package. Genes with |log2 fold change (FC)|> 1 and P < 0.05 were considered as DEGs. The intersection of DEGs and TSRGs were identified as DE-TSRGs. For functional enrichment analysis of these gene groups, we utilized Gene Ontology (GO) categories (biological process (BP), molecular function (MF), and cellular component (CC)) and KEGG pathway databases^18^, implemented in R.

### Immune infiltration and immune-related factors

The relationship between AMI and myocardial fibrosis is strongly influenced by immunity and inflammation. To evaluate the distribution of 22 immune cell types in each sample, we performed a comprehensive analysis of immune cell composition in AMI using the CIBERSORT method, which estimates the relative proportions of diverse immune cell types^19^. We also used Spearman coefficients to construct a correlation matrix among the proportions of the 22 immune cell subtypes. Moreover, we employed Pearson correlation analysis to substantiate the association between DE-TSRGs and immune cell infiltration.

### Machine learning methods and LASSO logistic regression

The Least Absolute Shrinkage and Selection Operator (LASSO) is a widely employed machine learning prediction technique that incorporates feature selection. In this study, we utilized LASSO logistic regression, implemented through the glmnet package in R, to effectively filter and identify diagnostic biomarkers associated with AMI^20^.

### The expression analysis, ROC curve analysis

The receiver operating characteristic (ROC) curves were generated using the pROC package in RStudio, and the diagnostic performance of the selected DE-TSRGs was assessed using the Area Under the ROC Curve (AUC)^21^. AUC values ≥ 0.6 for DE-FRGs indicate diagnostic value in AMI, while a p-value < 0.05 signifies a statistically significant difference.

### Animals, MI model

This study adhered to NIH guidelines for animal care and was approved by the Hubei Medical University Animal Care and Use Committee. Male Sprague–Dawley rats (250-300 g) were supplied by the Experimental Animal Centre of Hubei Medical University. AMI model was induced by permanent ligation of the left-anterior-descending (LAD) coronary artery to investigate MEOX1's pro-fibrosis effects on MI. Rats were anesthetized using pentobarbital(50 mg/kg,ip) and then ventilated. AMI was induced by tying an 8–0 nylon suture around the LAD artery^22^. AMI was confirmed by observing a pale color on the left ventricle and ST-segment elevation on the ECG. After experiment, the hearts were harvested for histological examination following anesthesia with pentobarbital.

### Masson's trichrome, immunohistochemistry and immunofluorescence

Cardiac tissues were fixed in 4% Paraformaldehyde, embedded in paraffin, and sectioned for Masson's Trichrome and immunohistochemistry staining. Paraffin sections were de-waxed, blocked with 5% normal horse serum in 0.1% BSA/PBS, and incubated overnight at 4 °C with goat anti-MEOX1 (1:200, ab105349, abcam). Sections were then stained with fluorescent secondary antibodies, DAPI, and mounted.

### In vitro angiogenesis experiment

In the in vitro angiogenesis experiment, materials including Matrigel, 96-well plates, and EP tubes were pre-cooled overnight^23^. Two repeated holes were created in each group, filled with 50 µL of Matrigel glue, and transferred to a cell incubator for solidification. Virus-infected cells were digested and 100 µL of cell suspension was added to each well. The plate was incubated for 6 h, followed by microscopic examination to count rings and nodes. Tubular structures were analyzed using Image J^24^. HUVEC cells and adenoviruses (Ad-GFP, Ad-MEOX1, Ad-shMEOX1) were supplied by the Clinical Medical Research Institute of Hubei University of Medicine.

### Cell Immunofluorescence

HUVEC cells were inoculated in 75 cm^2^ culture dish and cultured with high glucose DMEM containing 10% fetal bovine serum (FBS) at 37 °C and 5% CO_2_. When cells confluence reached 70–80%, the culture medium was replaced with high glucose DMEM containing 2% horse serum to induce HUVEC cells differentiation. Immunofluorescence staining of CD31 (ab76533, 1:500, abcam), sarcomeric actin (ab7817, 1:500, abcam) were used to evaluate endothelial-mesenchymal transition. Additionally, the tubular situation was analyzed with Image J^24^. We established a hypoxia-reoxygenation(H/R) model in HUVECs, wherein the cells were subjected to 6 h of hypoxia followed by 2 h of reoxygenation. Subsequently, we extracted RNA from these cells to assess the expression of DE-TSRGs^25^.

### Quantitative reverse transcription PCR (RT-qPCR)

The mRNA expression levels of MEOX1 were quantified using RT-qPCR, with each sample being analyzed in triplicate to ensure accuracy. The primer sequences for the genes of interest are as follows: for MEOX1, forward (F): 5′-CTAGGGCCTTTGCTCCCACACT-3′ and reverse (R): 5′-GCCAAGAGACGCTGAGAAGCAGTA-3′; for GAPDH, forward (F): 5′-GCTCATTTCCTGGTATGACAACG-3′ and reverse (R): 5′-AGGGGTCTACATGGCAACTG-3′.

### Statistical analysis

Statistical analysis was conducted using R language and GraphPad prism 9. Images were processed with ImageJ, Adobe Photoshop, and Adobe Illustrator CS2 software.The data are presented as mean ± standard error of the mean (SEM). Normally distributed variables were compared by Student’s *t* test, or one-way analysis of variance (ANOVA) followed by Bonferroni correction for multiple comparisons. Chi-square test was used to analyze the counting data. P < 0.05 was considered statistically significant. For correlation analysis, Pearson correlation coefficients were calculated.

### Ethical approval

This study is conducted and reported in compliance with the ARRIVE guidelines. Ethical approval and consent for participation were obtained for all animal experiments, including the euthanasia procedures for rats. The experiments were meticulously carried out in accordance with the guidelines stipulated in the Care and Use of Laboratory Animals by the National Institutes of Health.

## Results

### Identifcation of DE-TSRGs in AMI

**Figure 1:**
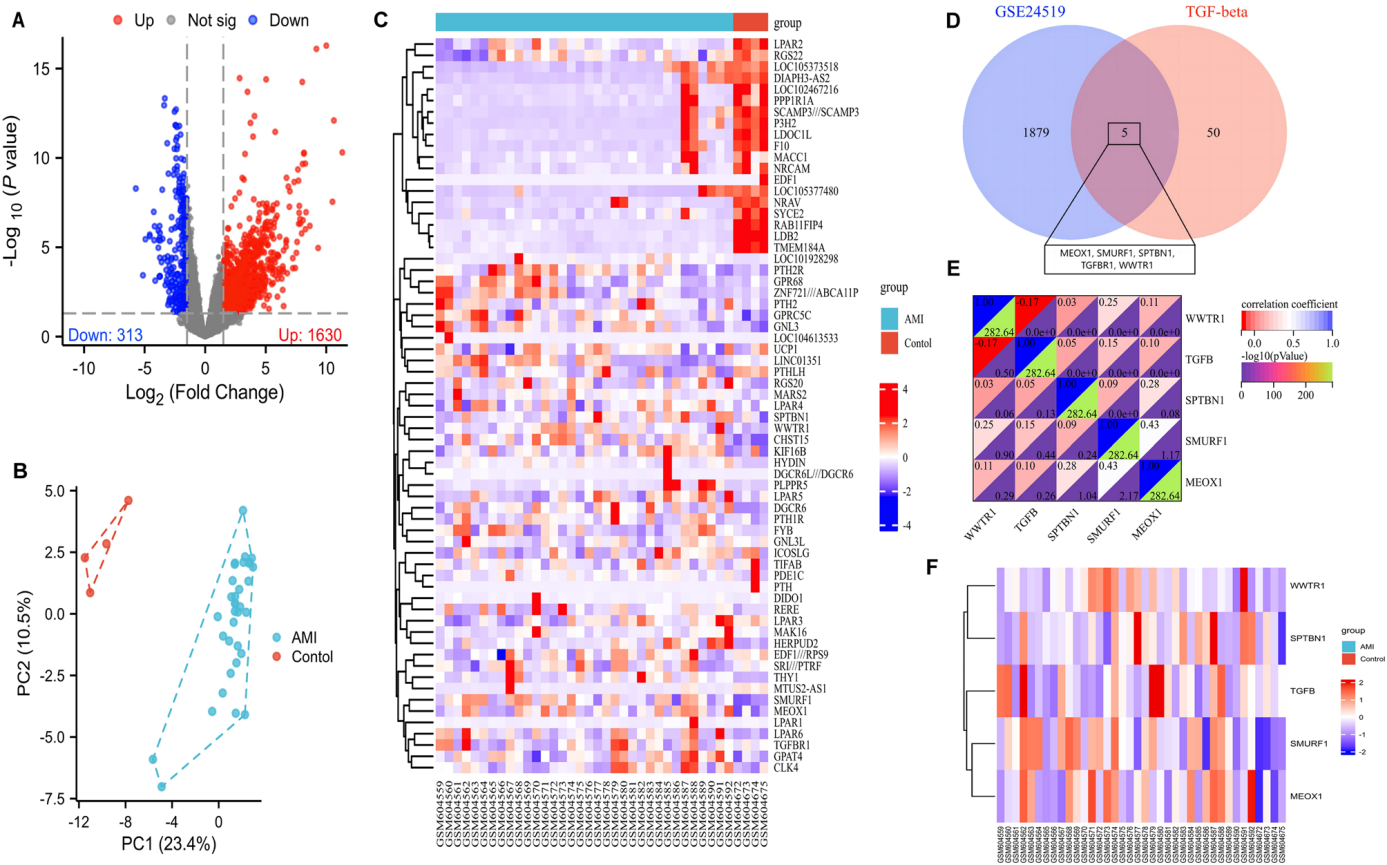
Identifcation of DE-TSRGs in the studied dataset. (**A**) The volcano map of DEGs. (**B**) Principle component analysis (PCA). (**C**) Heat map of DEGs. (**D**) 5 TSRGs were identifed as DE-TSRGs afer taking the crossover of DEGs and TSRGs. (**E**) Correlation analysis of the 5 DE-TSRGs. (**F**) Heat map showing 5 DE-TSRGs between AMI and control.

The overall study framework is depicted in a flow diagram (Sup. Fig. 1). Utilizing the GEO2R online tool, we identified 1884 DEGs from the dataset by comparing AMI samples with control samples. A PCA is illustrated in Fig. 1B. The volcano plot (Fig. 1A) and heatmap (Fig. 1C) collectively display the identified DEGs, highlighting their expression patterns and significance levels. To investigate TGF-β signaling pathway-related genes in AMI, we identified 55 TSRGs. After intersecting DEGs and TSRGs, we found 5 DE-TSRGs (Fig. 1D). The correlation and differential expression analyses of these 5 DE-TSRGs within the GSE24519 dataset are further detailed in Fig. 1E and F. Our analytical approach provides a focused examination of the TGF-β signaling pathway’s involvement in AMI, offering potential insights into the molecular underpinnings of the disease.

### Functional enrichment analysis of DE-TSRGs

**Figure 2:**
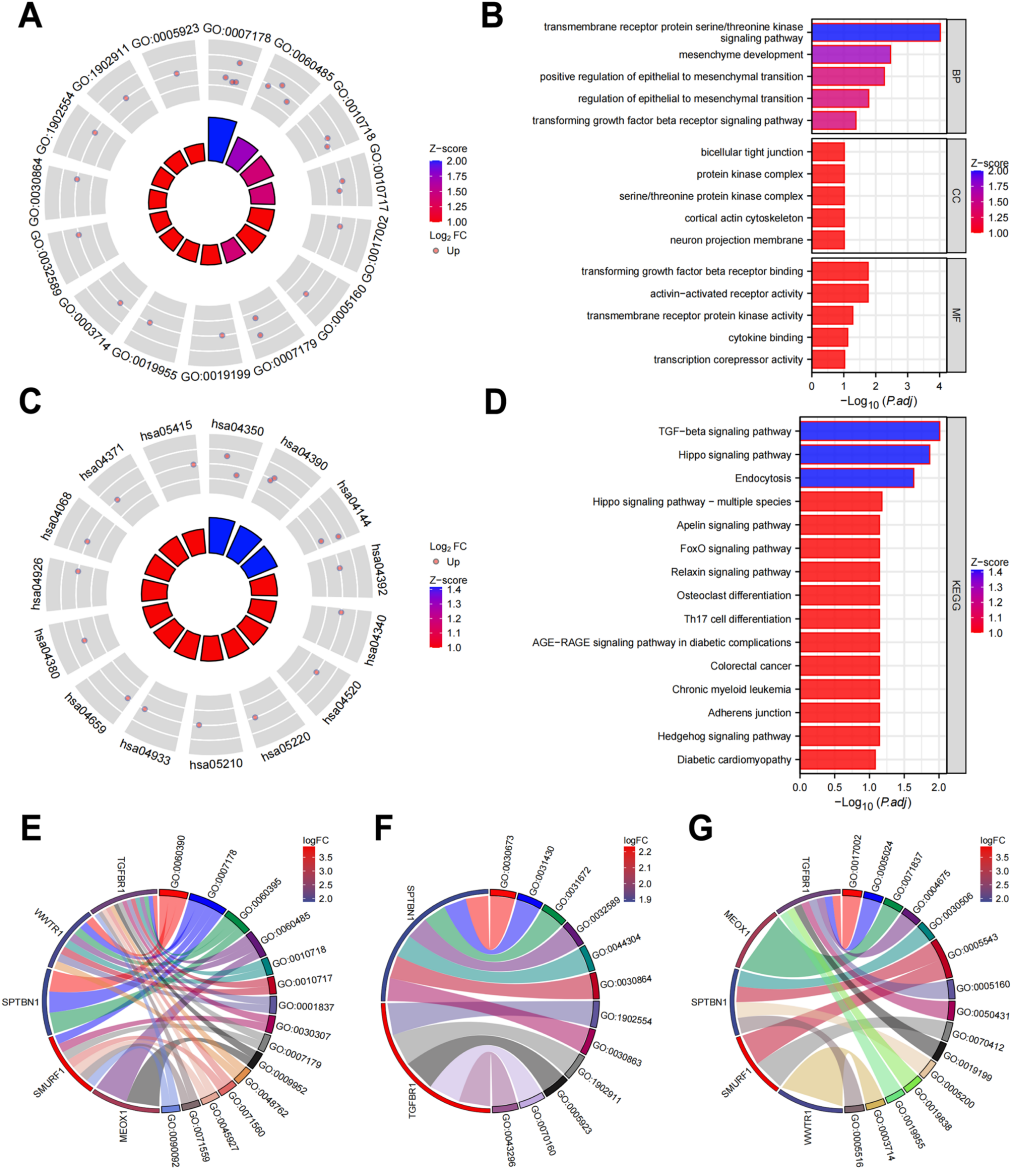
GO and KEGG enrichment analysis. (**A**,**B**) DE-TSRGs GO enrichment analysis. (**C**,**D**) DE-TSRGs KEGG analysis. (**E**–**G**) BP, CC, MF enrichment analysis.

To elucidate the biological implications of the DEGs identified in our study, we performed a functional enrichment analysis (Fig. 2A–D). GO analysis indicated that the DEGs' biological process changes were significantly enriched in SMAD protein signal transduction regulation and transmembrane receptor protein serine/threonine kinase signaling pathway (Fig. 2E). The cellular component changes in DE-TSRGs were notably concentrated in the axolemma, M band, and A band (Fig. 2F). In terms of molecular function, we observed significant enrichment in activin binding, transforming growth factor beta-activated receptor activity, and I-SMAD binding (Fig. 2G). KEGG pathway enrichment analysis revealed significant enrichment in TGF-β signaling, Hippo signaling, and Endocytosis pathways (Fig. 2C,D). These findings provide a deeper insight into the functional landscape of the DEGs, particularly in relation to the TGF-beta signaling pathway, and may help to identify potential therapeutic targets for acute myocardial infarction.

### Analysis of immune infiltration

**Figure 3:**
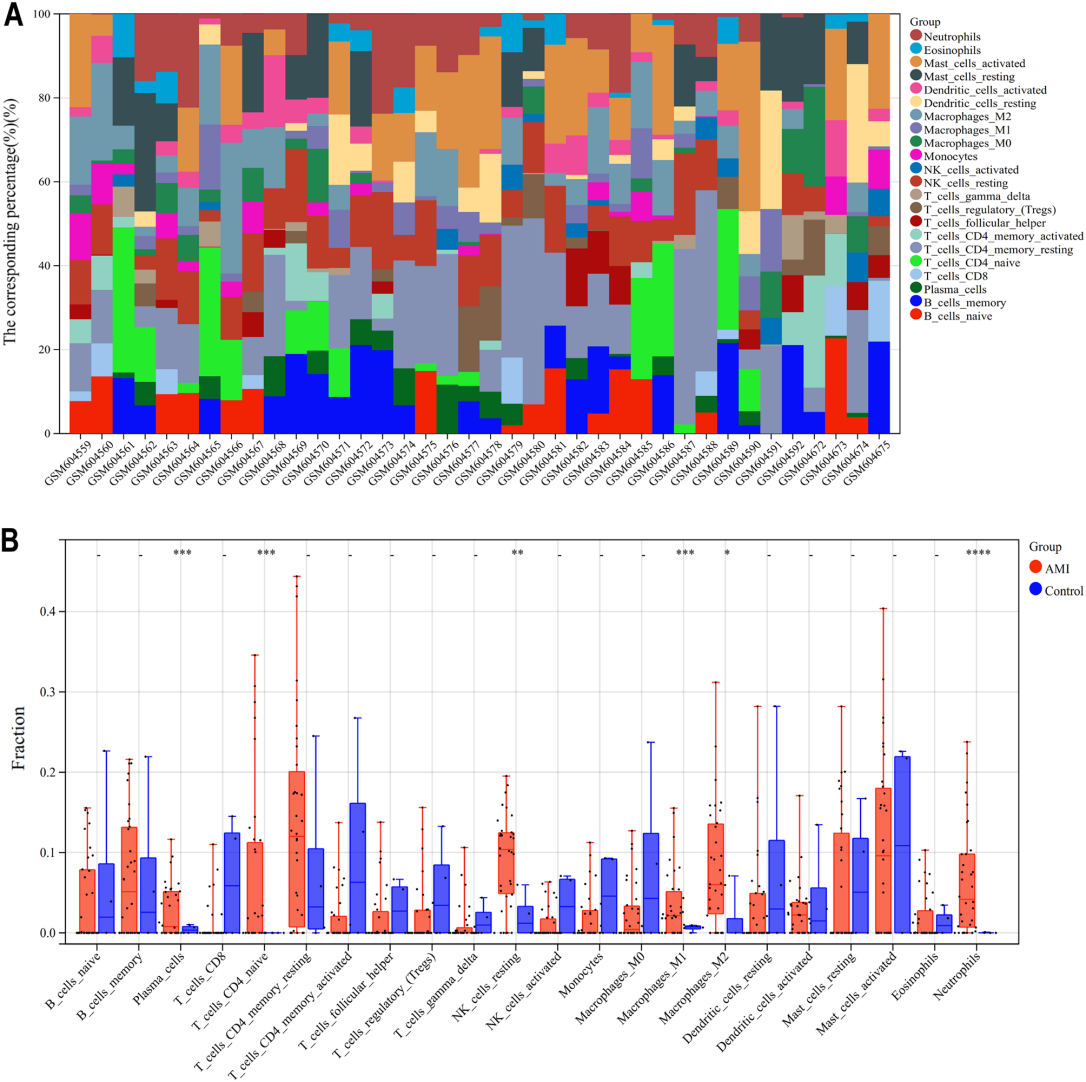
Infltration of immune-associated cells in healthy and AMI samples. (**A**) Relative percentage of 22 subpopulations of immune cells in each samples. (**B**) Differences in immunocell infiltration between AMI and control groups.

The CIBERSORT algorithm was employed to initially examine the proportion of 22 immune cell subsets in both AMI and control samples (Fig. 3A and B). Our results revealed a significant alteration in the immune cell composition in AMI compared to non-AMI controls, including neutrophils, macrophages, mast cells, dendritic cells, T cells, and NK cells (Fig. 3A). In the AMI group, neutrophils showed a marked increase in abundance. Additionally, there was a substantial elevation in the levels of M0 and M1 macrophages, naive CD4 T cells, resting NK cells, and plasma cells in the AMI group relative to controls. Conversely, the AMI group presented with reduced quantities of mast cells, dendritic cells, and naive B cells (Fig. 3B).These findings underscore the complexity of immune cell dynamics in the context of acute myocardial infarction and may provide valuable insights into the immunopathology of the condition.

### Correlation between DE-TSRGs and immune-infltrated cells in AMI

**Figure 4:**
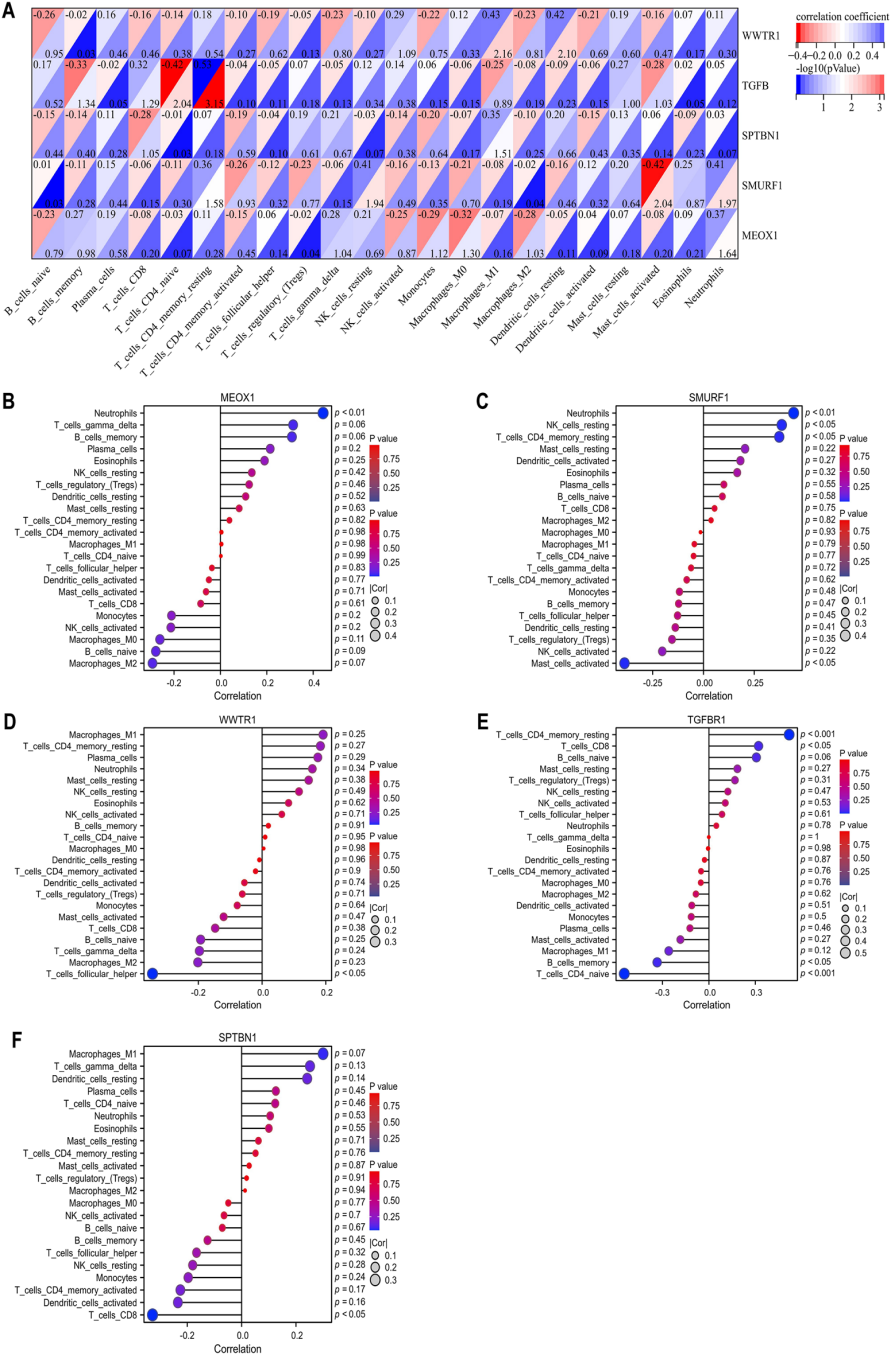
Correlation between DE-TSRGs and immune-infltrated cells in AMI. (**A**) Correlation matrix of DE-TSRGs. (**B**–**F**) The correlation analysis of DE-TSRGs and immune-infltrated cells, P < 0.05.

To elucidate the potential associations between the expression of DE-TSRGs and immune cell infiltration, we applied the Spearman correlation method, with the results presented in Fig. 4A. Notably, the strongest positive correlations were identified between MEOX1 expression and neutrophils (p value < 0.05) (Fig. 4B). The expression of SMURF1 was found to be significantly positively correlated with neutrophils, resting NK cells, and resting CD4 memory T cells, while it was negatively correlated with activated Mast cells (p value < 0.05) (Fig. 4C). Similarly, WWTR1 expression exhibited a negative correlation with helper follicular T cells (p value < 0.05) (Fig. 4D). Furthermore, TGFBR1 expression was positively correlated with the number of resting CD4 memory T cells and CD8 T cells, but negatively correlated with memory B cells and naive CD4 T cells (p value < 0.05) (Fig. 4E). Additionally, SPTBN1 expression levels showed a negative correlation with CD8 T cells (Fig. 4F).

Spearman correlation analyses were conducted on 22 immune cell types (Sup. Fig. 2), revealing a significant positive correlation between resting Mast cells and M0 Macrophages (correlation coefficient = 0.688, p value < 0.05). Additionally, a strong negative correlation was observed between resting Mast cells and activated Mast cells (correlation coefficient = − 0.808, p value < 0.05).These findings provide valuable insights into the intricate interplay between DE-TSRGs and immune cell infiltration in the context of acute myocardial infarction, potentially guiding future research into targeted therapies.

### Screening key DEGs by machine learning methods, LASSO logistic regression and ROC curves

**Figure 5:**
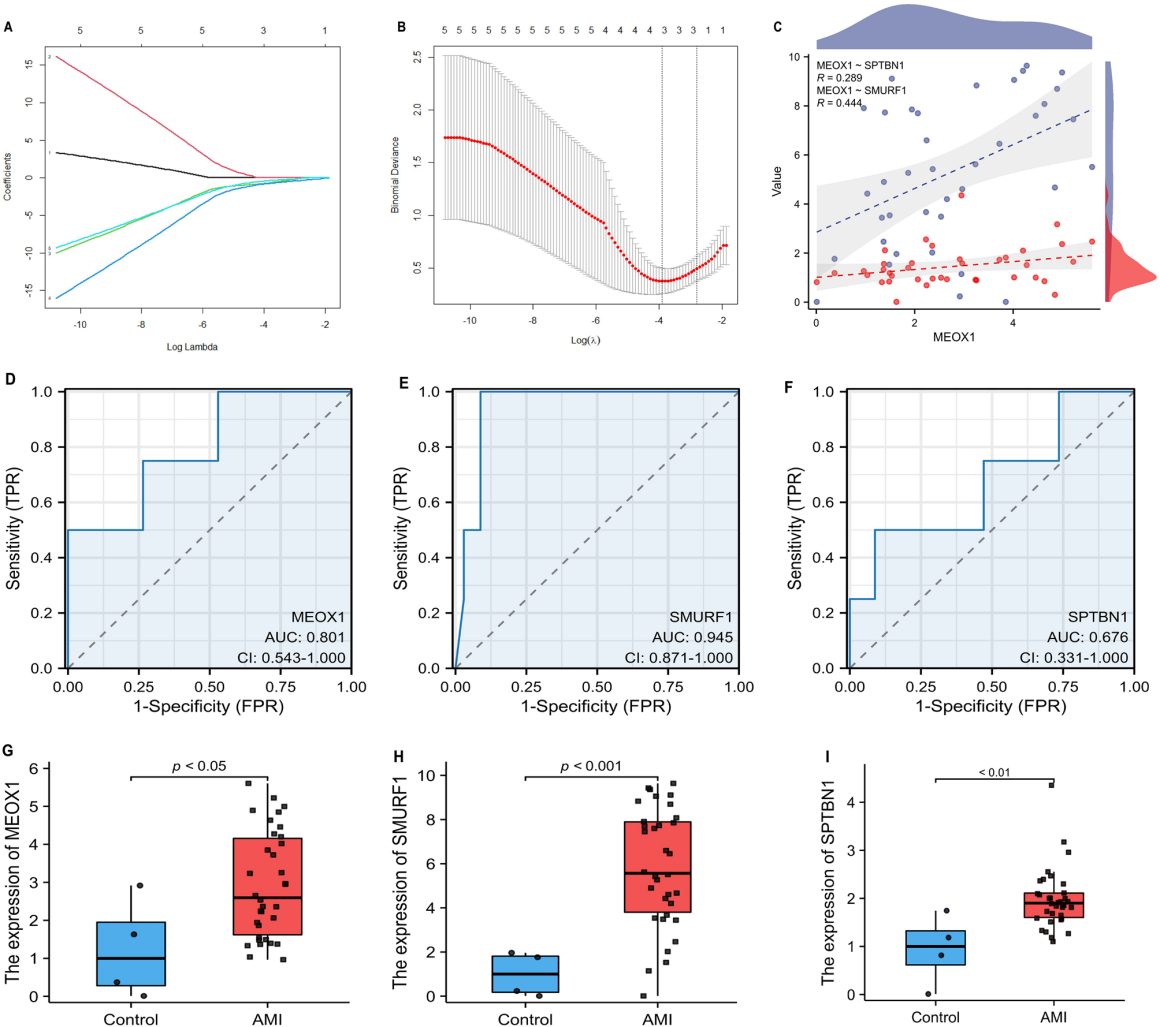
Analysis of hub DE-TSRGs. (**A**) Lasso regression of the variable selection. (**B**) The area of best λ values varies, the best variable numbers should be 3. (**C**) Correlation between MEOX1, SMURF1 and SPTBN1 in AMI. (**D**–**F**) ROC curves of hub gene expression in GSE24519. (**G**–**I**) Boxplot of hub gene expression in GSE24519.

The DE-TSRGs were selected through the utilization of the LASSO regression algorithm, employing the lambda value associated with the minimum mean error. This process led to the identification of three variables, namely MEOX1, SMURF1, and SPTBN1, as diagnostic biomarkers for AMI, as depicted in Fig. 5A–C. The rigorous selection process underscores the robustness of these biomarkers, which may hold substantial promise for enhancing the diagnostic accuracy of acute myocardial infarction.

To assess the diagnostic efficacy of MEOX1, SMURF1, and SPTBN1, ROC curves were constructed and the corresponding AUC were calculated. Consistent with expectations, MEOX1 and SMURF1 exhibited robust diagnostic performance in AMI, while SPTBN1 demonstrated suboptimal performance, with AUC values of 0.801, 0.945, and 0.676, respectively (Fig. 5D–F). The differential expression levels of these three key differentially expressed transcriptome signature-related genes (DE-TSRGs) in AMI compared to normal samples were depicted in Fig. 5G–I. Taken together, these findings further support the notion that MEOX1 and SMURF1 hold promise as potential biomarkers for AMI diagnosis. It is noteworthy that numerous studies have been conducted on the function of SMURF1, whereas there is a lack of literature regarding MEOX1 and AMI^26–29^. Consequently, in order to delve deeper into the potential implications of MEOX1 upregulation in AMI, we undertook a rat model of myocardial infarction to authenticate the impact of MEOX1 on AMI in vivo, as well as to investigate the underlying mechanisms.

### Increased expression of MEOX1 is associated with myocardial fibrosis in the AMI rats

**Figure 6:**
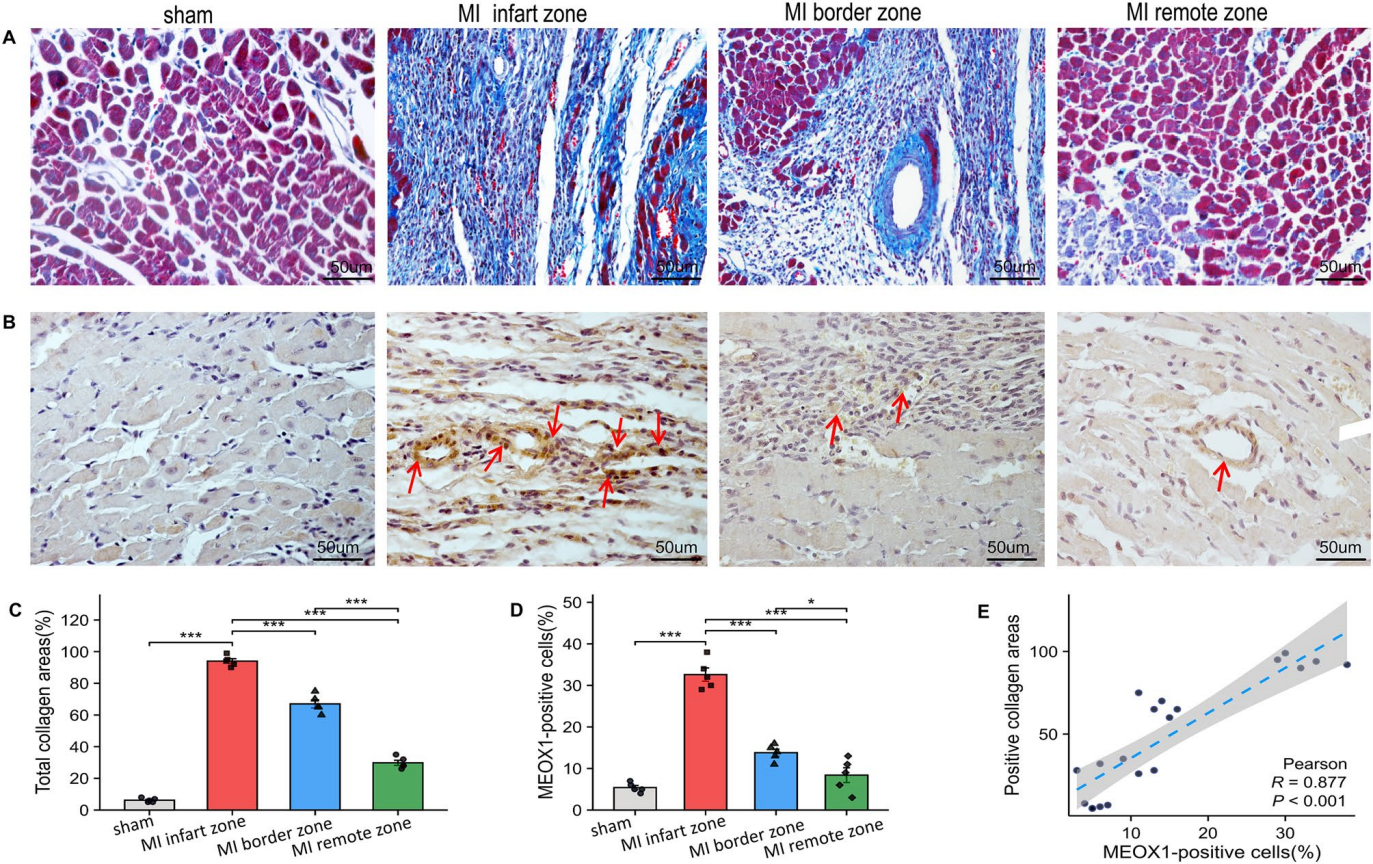
MEOX1 promotes myocardial fibrosis following AMI. (**A**) Typical Masson’s trichrome staining of left ventricular myocardium among the three groups. The blue areas indicate the collagen depositions. (**B**) Representative immunohistochemical staining of MEOX1 in ventricular myocardium among the four groups. (**C**) Quantitative statistics of positive collagen areas, (n = 5) per group, *P < 0.05 indicates statistical significance. (**D**) Quantitative analysis of MEOX1-positive cells among the three groups. *P < 0.05, n = 5. (**E**) Pearson correlation of MEOX1 with the positive collagen areas, (n = 5) per group, *P < 0.05 indicates statistical significance. Results are expressed as the mean ± SEM. ns P > 0.05, *P < 0.05, **P < 0.01, ***P < 0.001.

**Figure 7:**
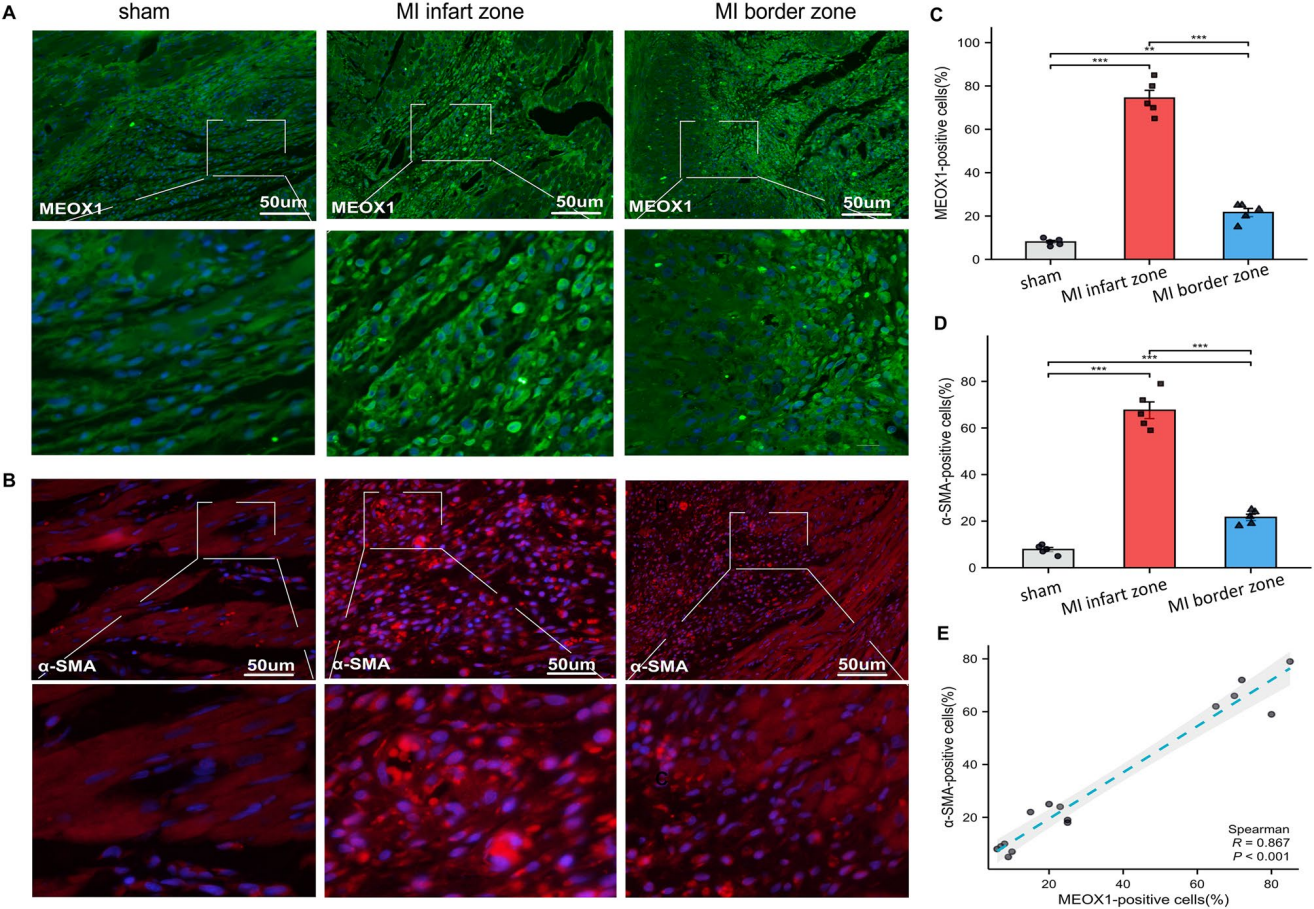
Expression of MEOX1 and α-SMA in ventricular myocardium among the three groups. (**A**) Representative immunofluorescence staining of MEOX1 in ventricular myocardium among the three groups. (**B**) Quantitative analysis of MEOX1-positive cells among the three groups. *P < 0.05, n = 5. (**C**) Representative immunofluorescence staining of α-SMA in ventricular myocardium among the three groups. (**D**) Quantitative analysis of α-SMA-positive cells among the three groups. *P < 0.05, n = 5. (**E**) Pearson correlation of MEOX1 with α-SMA, (n = 5) per group, *P < 0.05 indicates statistical significance. Results are expressed as the mean ± SEM. ns P > 0.05, *P < 0.05, **P < 0.01, ***P < 0.001.

In the AMI group, we observed S-T segment elevation on the ECG compared with the sham group, confirming the successful induction of AMI (Sup Fig. 3A,B). Furthermore, the messenger RNA (mRNA) levels of MEOX1 were validated by RT-qPCR (Sup Fig. 3C). Consistent with our expectations, Masson staining revealed increased collagen accumulation in the infarct, peri-infarct, and remote regions of the left ventricular myocardium in AMI rats (Fig. 6A,C). Immunohistochemical staining demonstrated a significant upregulation of MEOX1 expression in the heart tissues of AMI rats compared to the control group (Fig. 6B, Fig. 6D). Pearson correlation analysis showed a positive correlation between MEOX1 expression and positive collagen areas, indicating statistical significance (Fig. 6E). Immunofluorescence analysis confirmed the presence of high MEOX1 and α-SMA expression in these tissues (Fig. 7A–D), suggesting a significant positive correlation between MEOX1 expression and myocardial fibrosis post-AMI (Fig. 7E). These findings highlight the potential role of MEOX1 in the pathophysiology of AMI and its association with myocardial fibrosis.

### Meox1 induces myocardial fibrosis after AMI via EndoMT

**Figure 8:**
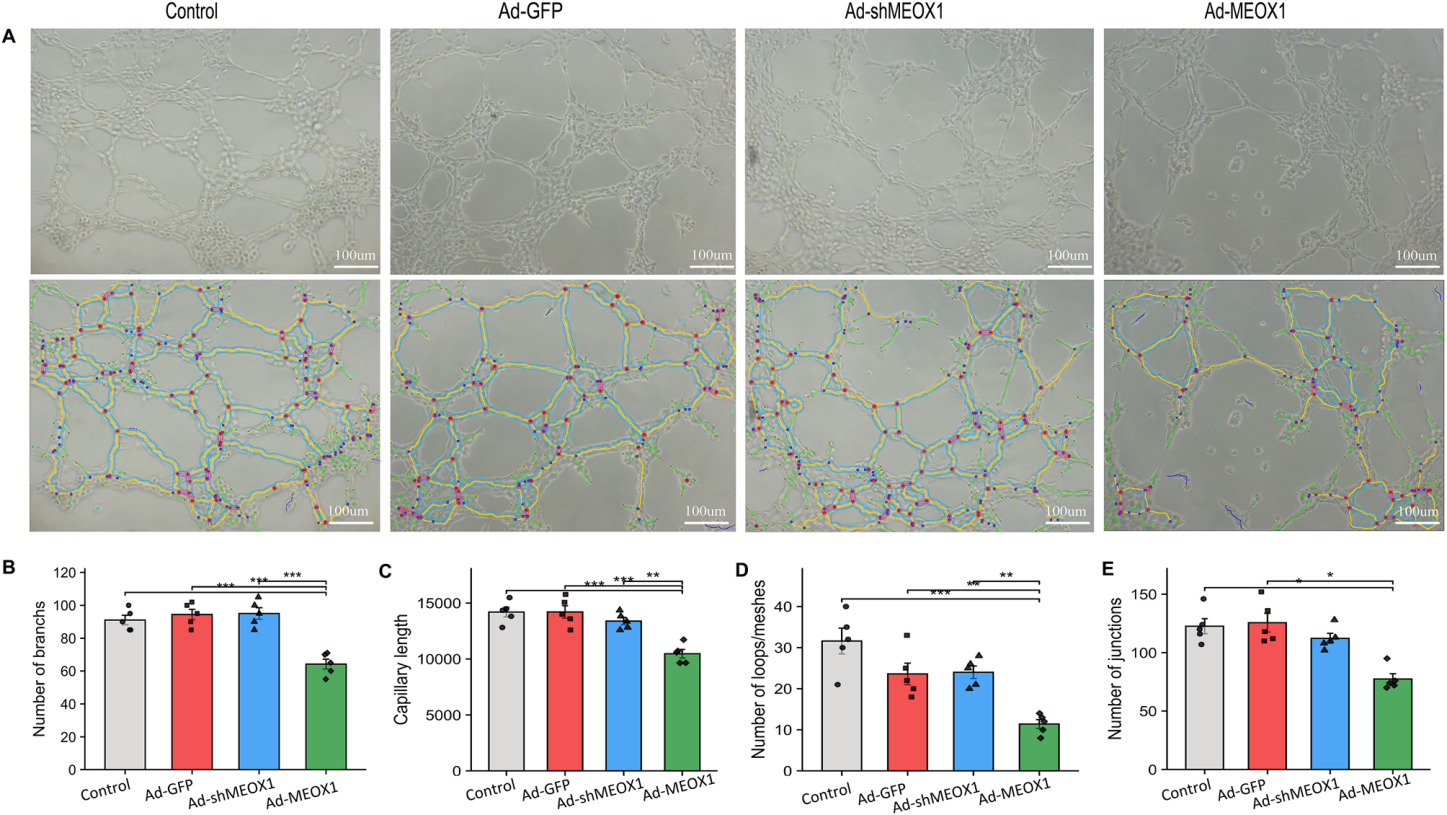
MEOX1 significantly inhibited the formation of tube-like structures by endothelial cells. (**A**) Representative images of angiogenesis experiment. (**B**–**E**) Quantitative analysis of angiogenesis experiment among the four groups. *P < 0.05, n = 5. Results are expressed as the mean ± SEM, and n represents the number of animals in each group. ns P > 0.05, *P < 0.05, **P < 0.01, ***P < 0.001.

**Figure 9:**
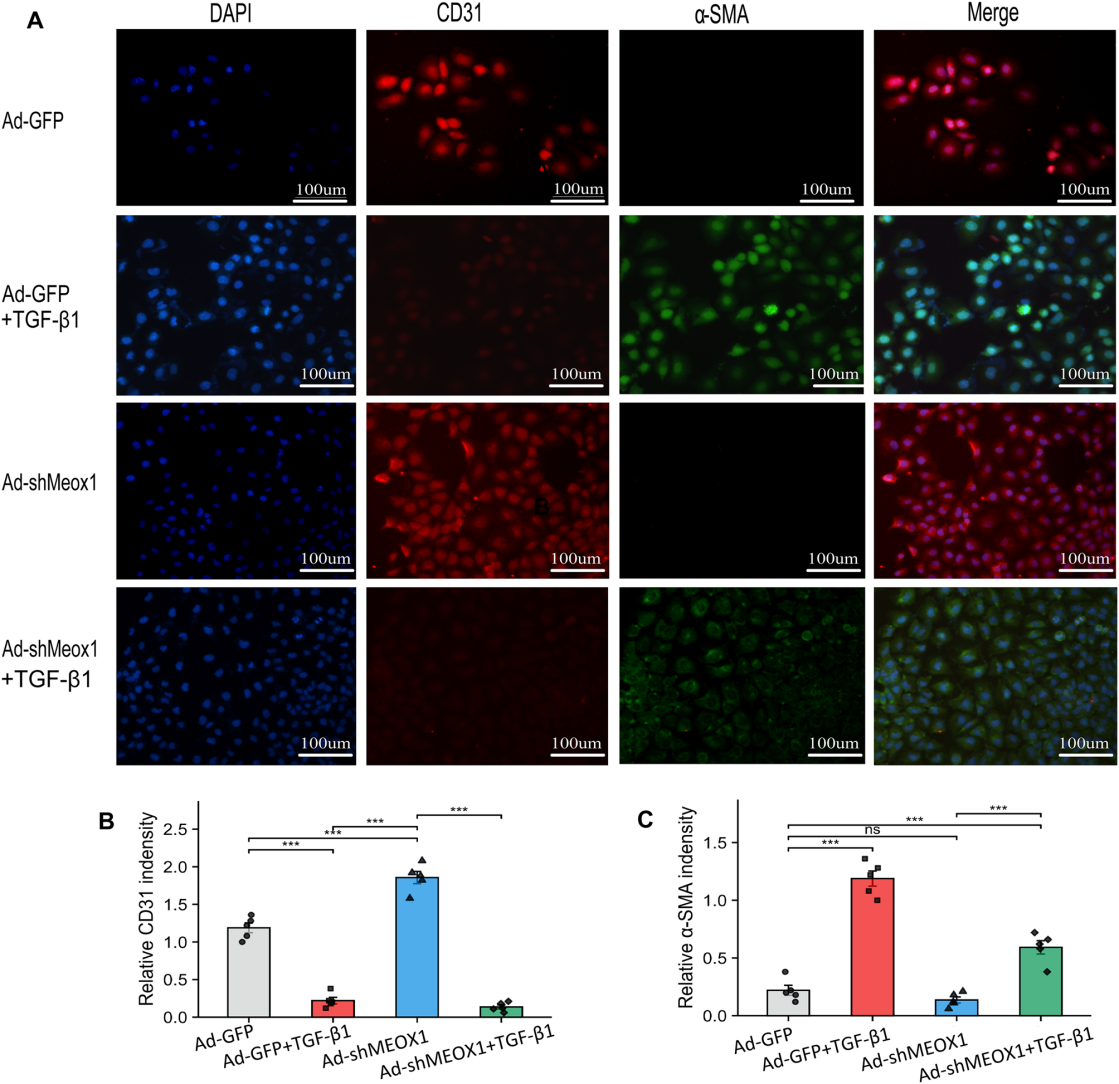
MEOX1 induces endothelial-mesenchymal transition. (**A**) Immunofluorescence results on HUVECs with the CD31 and α-SMA and its quantitative statistics (**B**,**C**), (n = 5) per group. *P < 0.05 indicates statistical significance. α-SMA, α-smooth muscle actin. Results are expressed as the mean ± SEM. ns P > 0.05, *P < 0.05, **P < 0.01, ***P < 0.001.

Following myocardial infarction, MEOX1 expression is predominantly observed in the microvascular endothelium of the infarct and peri-infarct myocardium RT-qPCR(Fig. 6B). Given the significant interstitial fibrosis in these areas (Fig. 7A and B), we hypothesize that MEOX1 may instigate myocardial fibrosis post-acute myocardial infarction via EndoMT. Specifically, we have successfully established a hypoxia-reoxygenation model in HUVECs, wherein the cells were subjected to 6 h of hypoxia followed by 2 h of reoxygenation, experiments demonstrated elevated MEOX1 expression levels in response to hypoxia-reoxygenation (Sup Fig. 3D). Furthermore, we evaluated the angiogenic potential of endothelial cells by assessing the formation of tube-like structures on Matrigel following transduction with Ad-MEOX1 or Ad-shMEOX1. Our findings indicate that overexpression of MEOX1 significantly hinders the formation of these structures (Fig. 8A–E), whereas MEOX1 knockdown via shRNA promotes their formation, (Fig. 8A and B). Despite the recognized importance of TGF-β signaling in endothelial-to-mesenchymal transition, the precise downstream targets of TGF-β-induced EndoMT remain elusive. Bioinformatics analysis suggests that MEOX1 may serve a crucial role as a downstream target of TGF-β-induced EndoMT. To investigate this further, Huvec cells were transfected with Ad-GFP and Ad-shMEOX1 and subsequently treated with 10 ng/ml TGF-β1 for 24 h, following a 24-h incubation period. MEOX1 knockdown resulted in a decrease in TGF-β-induced expression of the myofibroblast marker α-SMA and increased expression of CD31 (Fig. 9A–C). In summary, our study reveals that MEOX1 expression is upregulated following AMI and, as a downstream target of TGF-β1, MEOX1 can inhibit angiogenesis and promote cardiac fibrosis post-AMI in vitro via EndoMT induction.

## Discussion

Prior research has established the pivotal role of myofibroblasts in the progression of severe cardiac fibrosis post-myocardial infarction^30–32^. Myofibroblasts can stem from diverse sources, including the proliferation of dormant fibroblasts within the tissue, the activation and subsequent migration and accumulation of CD34 + fibrocytes from bone marrow, or via epithelial-mesenchymal transition on epithelial cell surfaces^33–35^. Additionally, myofibroblasts can also originate from other cell types, including adventitial cells, lipid adipocytic cells, or activated macrophages^36^. Recent studies have identified fibres as an additional source of activated myofibroblasts in degenerative diseases, specifically via the endothelial-to-mesenchymal transition mechanism^37^.This process entails the detachment and morphological transformation of endothelial cells, leading to the acquisition of a mesenchymal phenotype characterized by elongated and spindle-shaped morphology^38^. These transformed cells lose their unique endothelial molecular markers, such as CD31/PECAM-1 and von Willebrand factor (vWF), and VE-cadherin expression. Concurrently, mesenchymal cell markers α-SMA, vimentin, and type I collagen are upregulated^39^.The activation of TGF-β initiates endogenesis, yet the intricate molecular mechanisms and intracellular cascades responsible for the phenotypic transition from endothelial to mesenchymal cells remain highly complex and not fully elucidated^40,41^.

The MEOX gene, specifically MEOX1, is crucial for embryonic development and the formation of body segments. In fibroblasts, the scATAC-seq data revealed that the transcription factor MEOX1 exhibited a significant increase in activity following TAC surgery^16^. Previous research has demonstrated the role of MEOX1 in organ development, modulation of cell cycle states, and induction of senescence in endothelial cells^14–17^. It has been established that MEOX1 and MEOX2 have overlapping functions in endothelial cells. However, the potential involvement of MEOX1 in cardiac fibrosis and fibroblast activation remains unexplored in prior investigations^14,42^.

The findings of this study shed light on the potential role of MEOX1 in the pathophysiology of AMI and subsequent vascular remodeling. The specific localization of MEOX1 in the microvascular endothelium of the infarcted region and margins, coupled with its upregulation in response to ischemia and hypoxia, supports its involvement in the pathological state following AMI. The observed impact of MEOX1 on neovascularization in endothelial cells further suggests its potential contribution to vascular remodeling post-AMI. The inhibition of neovascularization upon MEOX1 overexpression and promotion of neovascularization with MEOX1 knockdown indicate a regulatory role for MEOX1 in endothelial cell phenotypic changes and vascularity. Moreover, the proposed involvement of MEOX1 in stimulating endothelial cells to undergo a phenotypic transition aligns with its potential role in the progression of fibrosis within the infarcted area. These findings offer novel insights into the mechanisms underlying cardiac fibrosis post-AMI and present MEOX1 as a potential target for therapeutic interventions aimed at mitigating fibrotic processes in the infarcted myocardium. Further research into the precise molecular mechanisms through which MEOX1 influences endothelial cell behavior and vascular remodeling post-AMI is warranted to validate its potential as a therapeutic target for cardiac fibrosis.

The positive correlation between MEOX1 expression and neutrophil infiltration in the context of AMI suggests potential biological significance. This correlation implies that MEOX1 may be involved in signaling pathways that mediate the recruitment of neutrophils to the infarcted myocardium, reflecting its role in the early inflammatory response to AMI. Additionally, MEOX1's association with the regulation of genes involved in inflammation and extracellular matrix remodeling aligns with the processes influenced by neutrophils post-AMI, indicating a coordinated response. Further mechanistic studies are warranted to elucidate the precise involvement of MEOX1 in neutrophil-mediated processes and its impact on cardiac repair post-AMI.

This study has identified several key findings: Firstly, MEOX1 has consistently been found to be differentially expressed in both the dataset of acute myocardial infarction and the TGFβ signaling pathway. Secondly, MEOX1 has been associated with ventricular structural remodeling and the onset of cardiac dysfunction, particularly characterized by the presence of ventricular interstitial fibrosis. Thirdly, MEOX1 has been observed to regulate endothelial properties and induce mesenchymal transition. Lastly, the induction of myocardial fibrosis after acute myocardial infarction is attributed to the action of MEOX1 through the process of EndoMT. In conclusion, the diagnostic and therapeutic implications of MEOX1 inhibition in the context of EndoMT following AMI hold significant promise for advancing personalized medicine and improving patient outcomes. The potential use of MEOX1 as a diagnostic biomarker and its role in guiding targeted therapeutic interventions underscore its potential to impact future treatment approaches for AMI.

However, this study has several limitations that need to be addressed. Firstly, the inclusion of a limited number of samples in the GEO dataset requires power analyses to assess and improve the sample size for validation purposes. Secondly, the evaluation of endothelial-to-mesenchymal transition and myocardial fibrosis could have been improved by using more accurate methodologies. Lastly, further verification is needed to establish the relationship between endothelial-to-mesenchymal transition, immune infiltration, and acute myocardial infarction. The progression of cardiac fibrosis involves various effectors and mechanisms^43^, such as autophagy and cardiomyocyte apoptosis, and it remains to be determined whether these mechanisms are involved in MEOX1-induced myocardial fibrosis.

## Conclusion

In this study, we conducted an assessment of the contributions of immune infiltration and EndoMT in the context of AMI. Notably, we identified MEXO1 as a gene associated with the TGF-β signaling pathway, which plays a crucial role in the development of myocardial fibrosis subsequent to AMI via EndoMT. The findings presented herein substantially enhance our comprehension of the molecular pathophysiology underlying AMI and provide innovative perspectives on the pathogenesis of AMI, as well as potential targets for therapeutic intervention.

### Supplementary Information

Supplementary Figures.

## Data Availability

The datasets supporting the conclusions of this article are available in the GEO (Gene Expression Omnibus) (https://www.ncbi.nlm.nih.gov/geo/); TGF-β signaling pathway-related genes were obtained from the MSigDB (HALLMARK_TGF_BETA_SIGNALING) (https://www.gsea-msigdb.org/gsea/msigdb/human/geneset/HALLMARK_TGF_BETA_SIGNALING.html) and PubMed (https://pubmed.ncbi.nlm.nih.gov/34163071/). Further inquiries can be directed to the corresponding author.

## Abbreviations

AMI: Acute myocardial infarction
EndoMT: Endothelial-mesenchymal transition
ECM: Extracellular matrix
TAC: Transverse aortic constriction
GEO: Gene expression omnibus
AUC: Area under the curve
TGF-β: Transforming growth factor β
BP: Biological process
CC: Cellular component
MF: Molecular function
DEGs: Diferentially expressed genes
TSRGs: TGF-β signaling pathway-related genes
DE-TSRGs: Differentially expressed TGF-β signaling pathway-related genes
LASSO: Least absolute shrinkage and selection operator
