# Supplementary material for: Analysis of immunoinfiltration and EndoMT based on TGF-β signaling pathway-related genes in acute myocardial infarction
Source: Sci Rep. 2024 Mar 2;14:5183. doi: 10.1038/s41598-024-55613-5 (PMC10908777; doi:10.1038/s41598-024-55613-5)
Supplement: Supplementary file 1 — Supplementary Figures. [file 41598_2024_55613_MOESM1_ESM.docx]

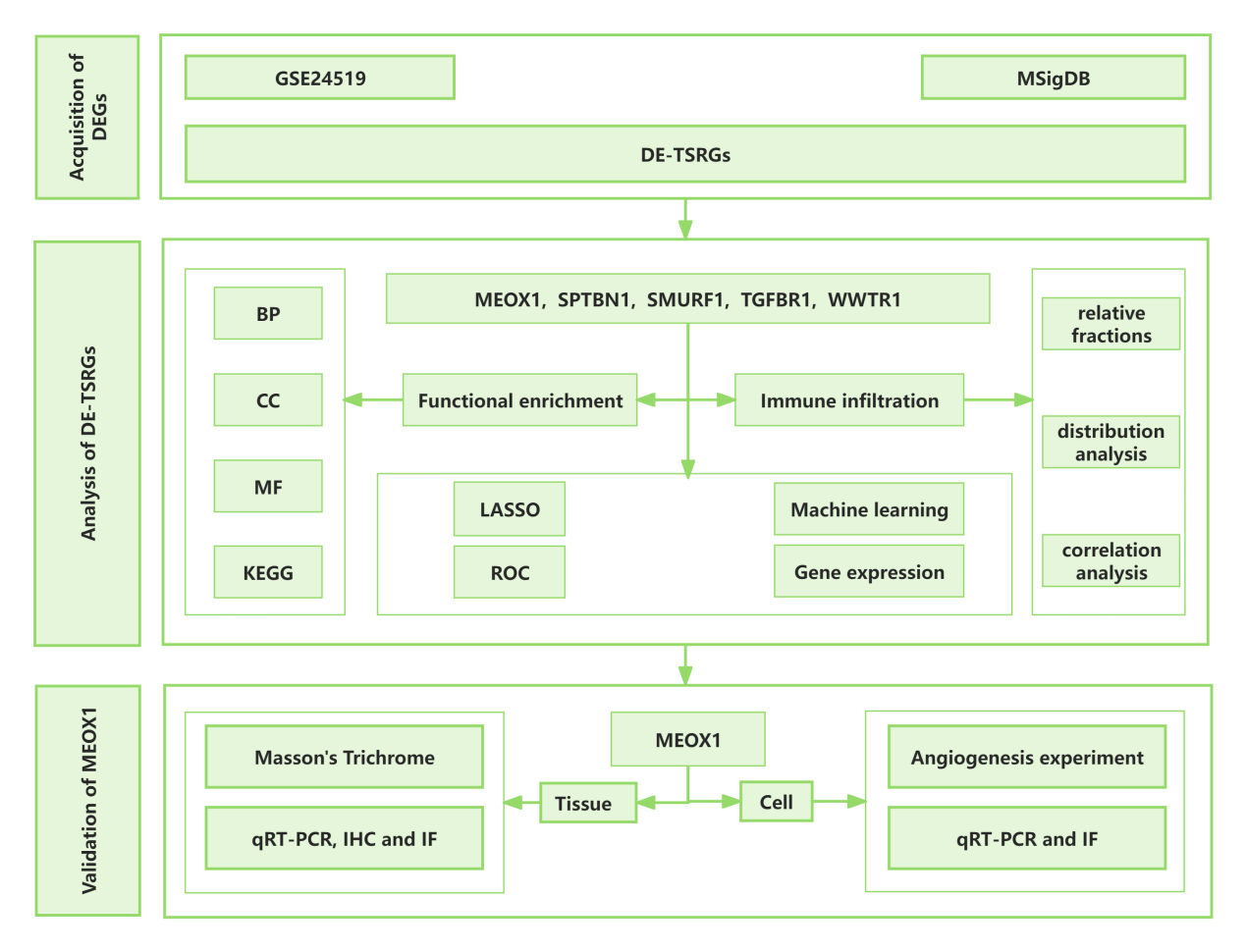


**Sup. Figure 1**. Flowchart summarizing overall bioinformatics analyses performed in this study to explore the biological characteristics of AMI.

**Abbreviations**: GO, Gene Ontology; KEGG, Kyoto Encyclopedia of Genes and Genomes;BP, biological process; MF, molecular function; CC, cellular component; DE-TSRGs, differentially expressed TGF-β signaling pathway-related genes; RT-qPCR, Quantitative Reverse Transcription PCR.


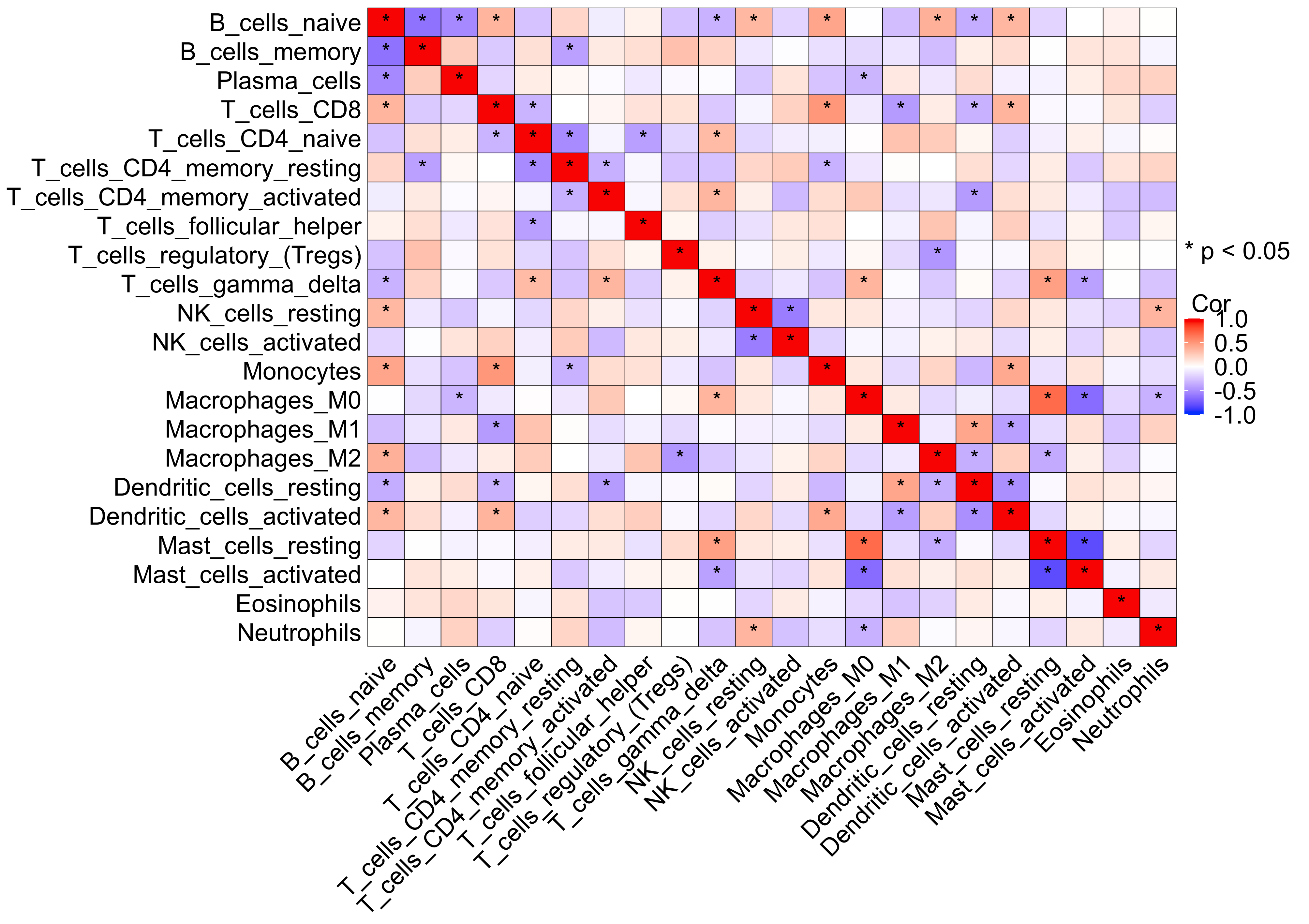


**Sup. Figure 2**. Correlation analysis. Red indicates a positive correlation and Blue indicates a negative correlation; the higher is the absolute value, the stronger is the correlation between immune cells. *P<0.05.


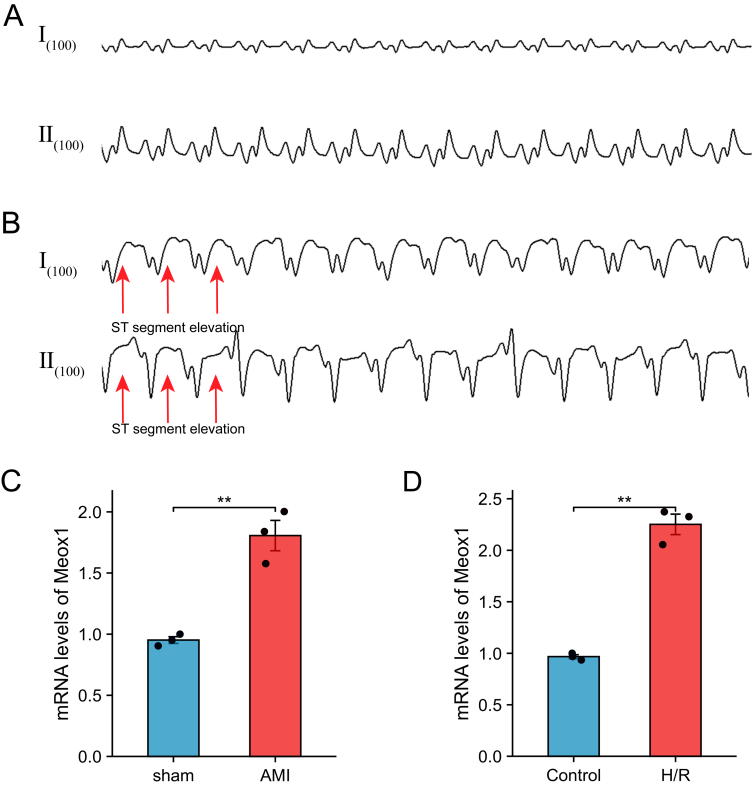


**Sup. Figure 3**. ECG and RT-qPCR. (A) ECG after sham surgery showing no ST segment elevation at lead I and II lasting over 30 min. (B) ECG after AMI showing ST segment elevation at lead I and II lasting over 30 min, (n=5) per group. (C) Measurement of MEOX1 mRNA levels by RT-qPCR in the AMI and sham groups. (D) Measurement of MEOX1 mRNA levels by RT-qPCR in the H/R and control groups. *P<0.05 indicates statistical significance. α-SMA, α-smooth muscle actin. Results are expressed as the mean±SEM. ns P > 0.05, *P < 0.05, **P < 0.01, ***P < 0.001. AMI indicates acute myocardial infarction model; H/R refers to the Hypoxia-Reoxygenation model in HUVECs.
